# Carpenter on Alcohol

**Published:** 1854-04

**Authors:** 


### CARPENTER ON ALCOHOL.

Dr. Carpenter has placed himself at the head of English physiologists by his various writings, and now is entitled to take the highest rank among the advocates of temperance. The volume before us is a temperance tract, and we are quite safe in saying that no work on that prolific subject was ever written with the same ability. As an exposition of the effects of alcohol on the human system, it is complete. All the branches of the subject are fully and most ably discussed. His argument is irresistible. No one can peruse his pages without giving a prompt and entire consent to his conclusions. His work is calculated to produce great good by its array of facts, and its high scientific character, and we are persuaded that we could not render a greater service to society than by promoting its circulation. It ought to be read by every physician. The author says that upwards of two thousand of his professional brethren—some of them among the most eminent in England—have signed the following certificate:—

"We, the undersigned are of opinion: 1st. That a very large proportion of human misery, including poverty, disease and crime, is induced by the use of alcoholic or fermented liquors as beverages.

"2. That the most perfect health is compatible with total abstinence from all such intoxicating beverages, whether in the form of ardent spirits, or as wine, beer, ale, porter, cider, &c., &c.

"3. That persons accustomed to such drinks may, with perfect safety, discontinue them entirely, either at once, or gradually after a short time.

"4. That total and universal abstinence from alcoholic beverages of all sorts would greatly contribute to the health, the prosperity, the morality, and the happiness of the human race."

There are few physicians, anywhere, who would not assent to the truth of these several propositions; but what are physicians doing to enforce and disseminate such doctrines? The greatest of all the evils by which human society is afflicted is, unquestionably the immoderate use of intoxicating liquors, and of the depth and intensity of the evil no class of the community are as constant or as competent witnesses as physicians. Physicians, therefore, ought to be in the van of the temperance movement. We are proud to say that they have been the leaders of this great reform, and it affords us the most sincere satisfaction to record the name of Dr. CARPENTER as prominent among this body of philanthropists.

This book is a small one and very cheap, and temperance societies cannot more efficiently promote their cause than by diffusing its doctrines. Messrs. BLANCHARD and LEA have preferred new claims to the respect and thanks of the community by issuing it in its present form, placing it within the reach of every one.

